# Non-contrast T1-mapping detects acute myocardial edema with high diagnostic accuracy: a comparison to T2-weighted cardiovascular magnetic resonance

**DOI:** 10.1186/1532-429X-14-42

**Published:** 2012-06-21

**Authors:** Vanessa M Ferreira, Stefan K Piechnik, Erica Dall’Armellina, Theodoros D Karamitsos, Jane M Francis, Robin P Choudhury, Matthias G Friedrich, Matthew D Robson, Stefan Neubauer

**Affiliations:** 1 Department of Cardiovascular Medicine, University of Oxford, John Radcliffe Hospital, Oxford, United Kingdom; 2 Stephenson Cardiovascular MR Centre, Libin Cardiovascular Institute of Alberta, Calgary, Alberta, Canada; 3 Department of Cardiology, Université de Montréal, Montréal, Quebec, Canada

**Keywords:** T1-mapping, ShMOLLI, Myocardial edema, Cardiovascular magnetic resonance, T2-weighted MRI

## Abstract

**Background:**

T2w-CMR is used widely to assess myocardial edema. Quantitative T1-mapping is also sensitive to changes in free water content. We hypothesized that T1-mapping would have a higher diagnostic performance in detecting acute edema than dark-blood and bright-blood T2w-CMR.

**Methods:**

We investigated 21 controls (55 ± 13 years) and 21 patients (61 ± 10 years) with Takotsubo cardiomyopathy or acute regional myocardial edema without infarction. CMR performed within 7 days included cine, T1-mapping using ShMOLLI, dark-blood T2-STIR, bright-blood ACUT2E and LGE imaging. We analyzed wall motion, myocardial T1 values and T2 signal intensity (SI) ratio relative to both skeletal muscle and remote myocardium.

**Results:**

All patients had acute cardiac symptoms, increased Troponin I (0.15-36.80 ug/L) and acute wall motion abnormalities but no LGE. T1 was increased in patient segments with abnormal and normal wall motion compared to controls (1113 ± 94 ms, 1029 ± 59 ms and 944 ± 17 ms, respectively; p < 0.001). T2 SI ratio using STIR and ACUT2E was also increased in patient segments with abnormal and normal wall motion compared to controls (all p < 0.02). Receiver operator characteristics analysis showed that T1-mapping had a significantly larger area-under-the-curve (AUC = 0.94) compared to T2-weighted methods, whether the reference ROI was skeletal muscle or remote myocardium (AUC = 0.58-0.89; p < 0.03). A T1 value of greater than 990 ms most optimally differentiated segments affected by edema from normal segments at 1.5 T, with a sensitivity and specificity of 92 %.

**Conclusions:**

Non-contrast T1-mapping using ShMOLLI is a novel method for objectively detecting myocardial edema with a high diagnostic performance. T1-mapping may serve as a complementary technique to T2-weighted imaging for assessing myocardial edema in ischemic and non-ischemic heart disease, such as quantifying area-at-risk and diagnosing myocarditis.

## Background

T2-weighted cardiovascular magnetic resonance (T2w-CMR) is the standard imaging technique for detecting acute myocardial edema in vivo [1]. It is used for differentiation of acute from chronic MI [2], assessment of the area-at-risk [3,4], myocarditis [5] and increasingly as a surrogate end-point in clinical trials [6].

T2w-CMR techniques are sensitive to an increase in myocardial water content and/or free water fractions [1,5-7]. Conventional T2w images are acquired using the short-tau inversion recovery (STIR) sequence at 1.5 T [8]. Limitations of this technique include signal drop-out, bright signals adjacent to the subendocardium due to slow-flow blood, image quality impairment in tachyarrhythmias, and long breath-holds [5,9]. Furthermore, there is need for a “normal” reference region of interest (ROI), either in remote myocardium, or in adjacent skeletal muscle for conditions with diffuse edema. The use of an ROI outside the myocardium typically requires the body coil to minimize signal inhomogeneity [5], which gives a lower SNR. In systemic conditions like viral myocarditis, the reference skeletal muscle may also be inflamed, leading to false negative results [5]. The bright-blood T2-weighted sequence ACUT2E [10] has been shown to be superior to dark-blood STIR in assessing acute myocardial infarction and area-at-risk [3], but has not been tested in subjects with diffuse or patchy edema using reference ROIs outside the myocardium.

T1-mapping may provide a quantitative means to detect myocardial edema without the need for reference ROIs. Each tissue type exhibits a characteristic range of normal T1 relaxation times at a particular magnetic field strength [11]. T1 and T2 times track closely together such that myocardial edema, which prolongs T2, also prolongs T1 [1,6,12]. Increased T1 values have been shown in ischemic tissue from dog hearts, which largely reflected increased free water content [13], and in humans with acute myocardial infarction [14,15]. However, the ability of T1-mapping to detect edema in the absence of infarction has not been systematically compared to T2w-CMR.

In this study, we compared T1-mapping using the novel ShMOLLI sequence [16], dark-blood and bright-blood T2w-CMR in the detection of acute myocardial edema. Patients presenting with Takotsubo cardiomyopathy (TCM) and regional edema in the absence of infarction were included. This allowed testing of the performance of each method in detecting both global and regional edema. We hypothesized that quantitative T1-mapping would be superior to T2-weighted methods that require reference ROIs.

## Methods

All subjects gave written informed consent and ethical approval was granted for all study procedures.

### Study population

Consecutive patients demonstrating CMR features of acute Takotsubo cardiomyopathy or acute myocardial edema without infarction were prospectively enrolled. The diagnosis of Takotsubo cardiomyopathy was based on the proposed Mayo Clinic criteria [17]: (1) an acute cardiac event presenting with chest pain and/or dyspnea; (2) transient systolic dysfunction with marked LV contraction abnormality (akinesis or dyskinesia of the LV apical and/or midventricular or basal segments) extending beyond a single coronary perfusion bed; (3) absence of significant (>50 %) obstructive coronary artery disease or angiographic evidence of acute plaque rupture; (4) new ECG abnormalities (either ST elevation or T wave inversion) or elevation in cardiac troponin level (upper limit Troponin I = 0.04 ug/L); (5) absence of phaeochromocytoma; and (6) absence of myocarditis or typical ischemic subendocardial/transmural LGE on CMR. Patients with acute myocardial edema without infarction not fulfilling the regional distribution criteria for TCM all had: (a) an acute cardiac event presenting with chest pain and/or dyspnea; (b) acute regional systolic dysfunction; (c) absence of significant (>50 %) obstructive coronary artery disease; (d) new ECG abnormalities (ST elevation or T wave inversion) and/or elevation in cardiac troponin level (upper limit Troponin I = 0.04ug/L); (e) absence of myocarditis or typical ischemic subendocardial/transmural LGE on CMR. Exclusion criteria included contraindications to CMR, known coronary artery disease, previous myocardial infarction, myocarditis or any chronic cardiac conditions. In addition, healthy volunteers with no prior cardiac history or known cardiac risk factors, not on cardiovascular medications and with a normal ECG underwent CMR, including cine, ShMOLLI T1-mapping, STIR and ACUT2E imaging.

### Cardiovascular Magnetic Resonance

CMR studies were performed using a 1.5 T MR system (Siemens Avanto, Germany). CMR scans assessed LV function, T1-mapping, edema and LGE, with matching short-axis images. T1-mapping was performed using the novel sequence ShMOLLI (Shortened Modified Look-Locker Inversion Recovery) [16]; dark-blood and bright-blood T2w-CMR were performed with the STIR [8], and the ACUT2E [10] sequences, respectively. All were acquired before administration of contrast agents. A 32-channel phased-array chest coil was used for all data acquisition, except for STIR imaging for which the body coil was used. LGE imaging was acquired using a T1-weighted phase-sensitive sequence [18] 6–10 minutes after intravenous administration of contrast agent (Gadodiamide, Omniscan, GE Healthcare, UK, total 0.13 mmol/kg body weight at 6 ml/s).

Typical imaging parameters for SSFP cine imaging were: voxel size 2.0x2.0x8.0 mm, TR/TE 39.6/1.12 ms, flip angle 55^o^, matrix 192x192; ShMOLLI T1-maps are based on 5-7 images with specific TI = 100-5000 ms, collected using SSFP readouts in a single breath-hold, typically: TR/TE = 201.32/1.07 ms, flip angle = 35°, matrix = 192x144, 107 phase encoding steps, interpolated voxel size = 0.9x0.9x8mm, cardiac delay time TD = 500 ms; 206 ms acquisition time for single image; STIR [5]: voxel size 1.9x1.5x10.0 mm, matrix = 256x166, effective echo time TE = 61 ms, effective repetition time TR = 2 RR intervals during breath-hold, flip angle 180^o^, echo spacing 6.74 ms, TI = 170 ms, dark blood thickness 200 %, dark blood flip angle 180^o^, turbo factor 25, echo trains per slice = 7; ACUT2E TSE-SSFP: voxel size 1.9x1.5x8.0 mm, matrix = 165x256, TR/TE = 229.70/1.78 ms, effective TE = 98 ms, flip angle 180^o^, T2 prep duration = 24 ms, segments = 33, 5 shots per slice, bandwidth = 781 Hz/Px); phase-sensitive inversion recovery sequence: voxel size 2.0 x 1.5 x 8.0 mm, matrix 144x256, TR/TE = 800.20/3.36 ms, flip angle 25^o^).

### Image Analysis

Matching short axis slices were compared across cine, ShMOLLI T1-mapping, STIR and ACUT2E imaging. Short axis images were manually contoured using an in-house software MC-ROI (programmed by SKP in IDL v.6.1, http://www.ittvis.com) to outline the endo- and epicardium, and then divided into 6 segments per slice using the anterior right ventricular-left ventricular insertion point as reference and for comparing segments amongst sequences.

### Cine images

Analysis of left ventricular ejection fraction was performed using Argus software (Version 2002B, Siemens Medical Solutions). Semi-quantitative assessment of wall motion abnormality was performed in MC-ROI. Segments were graded as: normal = 1, hypokinetic = 2, akinetic = 3, or dyskinetic = 4.

### ShMOLLI T1-maps

After T1-maps were generated, the LV myocardium was contoured and segmented as described. The average T1 values were calculated as the average of the T1 values of all pixels for that slice or segment using the in-house software MC-ROI as previously described [16].

### Quality assessment of T1-maps

As T1-maps are derived from non-linear estimation based on a sequential series of SSFP images acquired after an inversion pulse [16], factors such as off-resonance artifacts, breathing motion and poor T1 model fit may lead to falsely high or low T1 values. The presence of off-resonance artifacts and diaphragmatic movement was assessed by examination of the raw T1-weighted SSFP images. To assess for how well T1 model fitting was achieved for each T1-map, we used parametric maps of the goodness-of-fit (R^2^ maps; see Additional file 1 for further explanation and examples). R^2^ maps provide an additional means to identify artifacts and areas with potentially compromised T1 accuracy, which allowed critical assessment of the quality of T1-maps to increase the reliability of T1-mapping compared to some of the standard CMR modalities. Intra- and inter-observer variability of T1 measurements was assessed by two operators blinded to the clinical data; one slice of T1-map per subject was randomly pre-selected and analyzed for all subjects in the study.

### Dark-blood STIR images

Quantitative analysis was performed by comparing the LV myocardium in short axis against 2 reference regions of interest: (1) in remote myocardium, and (2) within adjacent skeletal muscle in the same slice, verified on a corresponding SSFP image. The T2 signal intensity (SI) ratio was calculated as: (1) T2 SI_myo:remote_ = SI_myocardium_/SI_remote myocardium_ and (2) T2 SI_myo:skeletal_ = SI_myocardium_/SI_skeletal__muscle_, as previously published [19]. Care was taken to exclude non-suppressed blood pool signal due to slow-flow adjacent to the subendocardium.

### Bright-blood ACUT2E images

Quantitative analysis was performed the same way as for STIR images on coil intensity normalized and distortion corrected images. If no suitable reference skeletal muscle were available on a slice (i.e. affected by artifacts), then a ROI with a mean SI and SD similar to that of skeletal muscle of the nearest neighbouring slice is placed on the index slice.

### Statistical analysis

Results are presented as interindividual mean ± SD with differences considered significant when p is <0.05 as assessed by unpaired 2-tailed Student T-tests. Receiver operator characteristic (ROC) analysis was performed to identify cut-off values of T1 and T2-weighted methods for their ability to distinguish between normal and affected myocardial segments. Myocardial segments in patients were considered to be affected by edema if there were acute wall motion abnormalities (wall motion score >1) in this clinical population as defined above, while segments in controls with normal wall motion were considered unaffected. As patient segments with normal wall motion may or may not be affected by edema, these segments were not included in the ROC analyses in order to identify optimal cut-off values for the methods studied. Significance of ROC analyses was assessed using the method of Delong et al. [20] (MedCalc, version11.5.1.0, http://www.medcalc.org). Intra- and inter-observer reproducibility of T1 mapping measurements were assessed by using the Bland-Altman method [21].

## Results

### Study population characteristics

Patient characteristics are presented in Table 1. Twenty-one patients (2 males, 61 ± 10 years) and 21 age-matched healthy controls (8 males, age 55 ± 13 years) were included. Patients with Takotsubo cardiomyopathy had a mean Troponin I of 8.26 ug/L (0.84-36.80 ug/L) and patients with regional edema had a mean Troponin I of 4.28 ug/L (0.15-12.69 ug/L). Ten out of the 12 patients with Takotsubo cardiomyopathy and six out of the 9 patients with acute regional edema had a clear stress precipitant (emotional or physical), while the rest had no clear precipitant.

**Table 1:** Patient characteristics

**Patient #**	**Age (yrs)**	**Gender**	**Precipitating factor (symptoms)**	**Time to CMR (days)**	**EF (%)**	**Diagnosis**	**Wall motion abnormality**	TnI*	**STIR T2 SI**	**ACUT2E T2 SI**	**T1 (ms)**
1	79	F	ES (ACP, P)	4	51	Takotsubo	Mid-apical AK	3.11	1.88	2.37	1043
2	56	F	Sz (ACP)	7	50	Takotsubo	Mid-apical AK	15.00	1.95	1.97	1065
3	70	F	PS (ACP)	2	64	Takotsubo	Mid-apical HK/AK	4.63	2.04	1.94	1051
4	60	F	Nil (ACP)	2	71	Takotsubo	Apical severe HK	19.98	1.89	2.20	1044
5	63	F	PS (D)	0	59	Takotsubo	Mid ventricular AK	4.46	1.57	1.85	1001
6	58	F	PS (ACP, D)	1	46	Takotsubo	Mid-apical AK	6.35	2.12	1.97	1032
7	42	M	Sz (ACP)	1	37	Takotsubo	Mid-apical HK/AK	5.00	1.93	1.64	1047
8	52	F	ES (ACP)	1	79	Takotsubo	Mid inferolateral & anteroseptal	22.75	2.29	2.56	1055
9	73	F	ES (ACP)	1	55	Takotsubo	Mid-apical HK/AK	10.86	1.91	2.18	1075
10	47	F	ES (ACP)	5	52	Takotsubo	Mid-apical HK/AK	0.84	2.57	2.43	1179
11	62	F	ES (ACP)	1	34	Takotsubo	Mid-apical HK/AK	36.80	1.90	2.14	1111
12	77	F	Nil (ACP)	4	66	Takotsubo	Mid ventricular AK	5.18	1.67	2.35	1100
13	47	F	Nil (ACP)	3	65	Regional edema	Inferolateral AK	1.30	1.57	1.98	993
14	66	F	ES (ACP)	2	51	Regional edema	Anterior,antero-septal AK	11.40	1.49	1.86	984
15	65	F	Nil (ACP)	1	62	Regional edema	Mid-apical inferolateral AK	12.69	1.48	1.85	1015
16	57	M	PS (ACP, D)	0	75	Regional edema	Distal anteroseptal HK	0.15	1.38	1.69	917
17	78	F	ES (ACP)	2	58	Regional edema	Mid inferior, inferolateral, inferoseptal AK	0.85	1.94	2.17	1133
18	59	F	ES (ACP)	4	67	Regional edema	Mid-apical anterior & anteroseptal	5.83	2.15	3.16	1113
19	54	F	ES (ACP)	1	66	Regional edema	Mid septum	2.63	1.56	2.07	999
20	63	F	Nil (ACP)	6	67	Regional edema	Mid-apical anterior & anteroseptum	3.46	2.02	2.13	1234
21	55	F	PS (ACP)	6	81	Regional edema	Mid anterior/ anteroseptal	0.21	1.86	2.28	1048

*Upper limit <0.04 ug/L for TnI. ACP = acute chest pain; TnI = Troponin I; T2 SI = T2 signal intensity (myo:skeletal); EF = ejection fraction; D = dyspnea; P = palpitations; PS = Physical stress; ES = Emotionalstress; Sz = Seizure. HK = hypokinesis; AK = akinesis. None of the patients demonstrated significant late gadolinium enhancement or coronary stenosis.

### CMR Findings

The median duration between symptoms and CMR was 3 days (0–7 days). CMR findings are summarized in Tables 1 and 2. The mean LVEF for both patients with Takotsubo cardiomyopathy (61 ± 10 %) and regional edema (66 ± 9 %) were significantly lower than controls (75 ± 6 %; both p < 0.01). None had significant LGE.

**Table 2:** Mean myocardial T1 and T2 SI ratios per-subject according to groups and segmental wall motion

**ShMOLLI T1 value (ms)**	**Controls**	**Takotsubo**	**Regional edema**	**All patients**
				
Normal wall motion	944 ± 17	1026 ± 24 (p < 0.001)	1032 ± 86 (p < 0.02)	1029 ± 59 (p < 0.001)
Abnormal wall motion	n/a	1101 ± 78 (p < 0.001)	1135 ± 120 (p < 0.001)	1113 ± 94 (p < 0.001)
Group average	944 ± 17	1064 ± 51 (p < 0.001)	1051 ± 98 (p < 0.01)	1059 ± 73 (p < 0.001)
STIR T2 SI_myo:skeletal_	Controls	Takotsubo	Regional edema	All patients
Normal wall motion	1.52 ± 0.14	1.79 ± 0.25 (p < 0.01)	1.65 ± 0.22 (p = 0.14)	1.73 ± 0.24 (p < 0.001)
Abnormal wall motion	n/a	2.16 ± 0.34 (p < 0.001)	1.96 ± 0.48 (p = 0.05)	2.09 ± 0.40 (p < 0.001)
Group average	1.52 ± 0.14	1.98 ± 0.26 (p < 0.001)	1.72 ± 0.28 (p = 0.08)	1.87 ± 0.29 (p < 0.001)
ACUT2E T2 SI_myo:skeletal_	Controls	Takotsubo	Regional edema	All patients
Normal wall motion	1.77 ± 0.19	1.93 ± 0.26 (p = 0.09)	1.97 ± 0.23 (p = 0.04)	1.95 ± 0.24 (p < 0.02)
Abnormal wall motion	n/a	2.34 ± 0.37 (p < 0.001)	2.49 ± 0.50 (p < 0.001)	2.40 ± 0.41 (p < 0.001)
Group average	1.77 ± 0.19	2.13 ± 0.27 (p < 0.001)	2.08 ± 0.29 (p < 0.02)	2.11 ± 0.27 (p < 0.001)

p values in brackets refer to the significance of the difference compared to values from normal controls.

**Figure 1:**
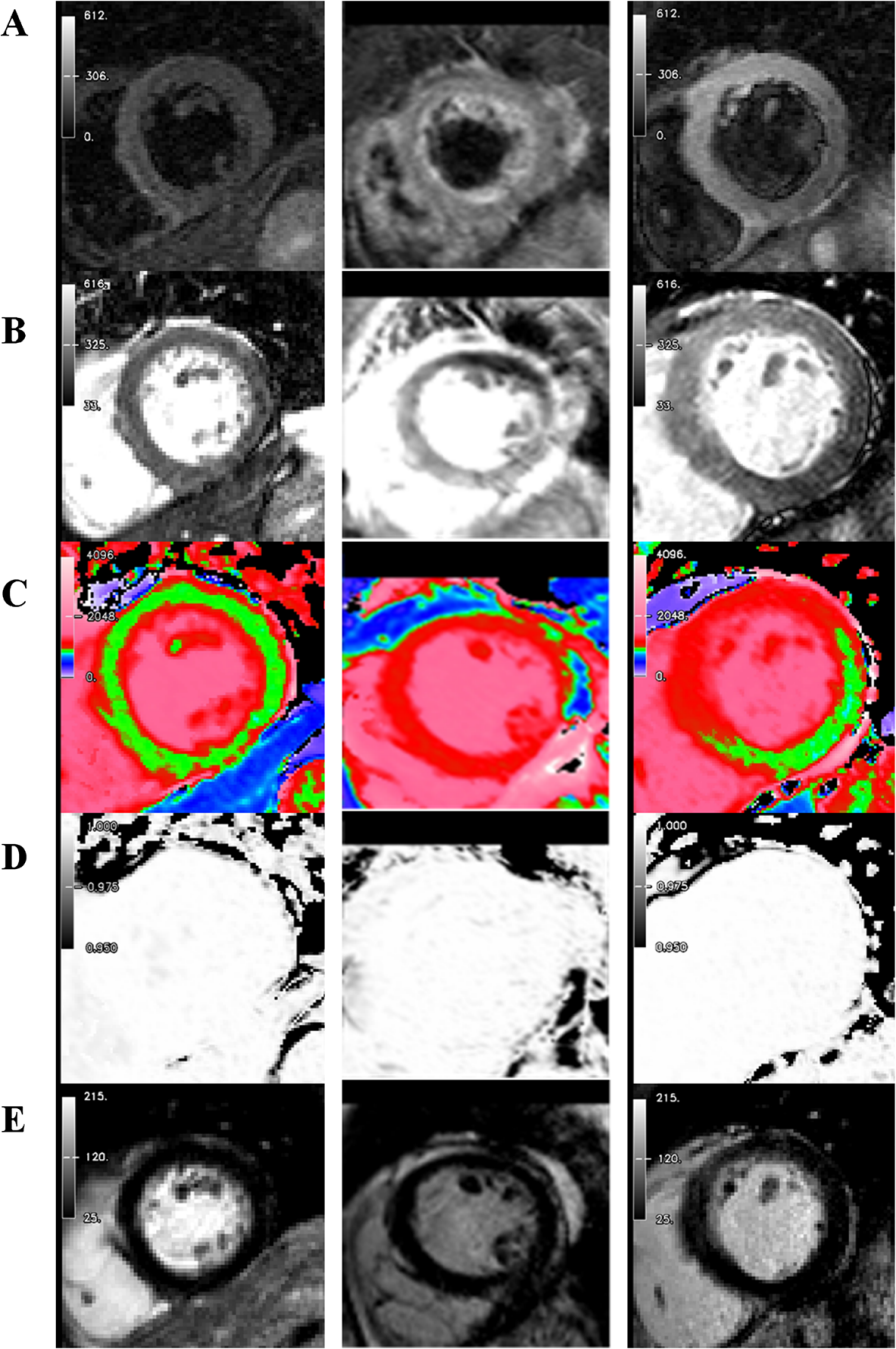
**CMR imaging modalities used for comparison – representative images in short-axis slices from: (*Left panel*) Normal volunteer (*Middle panel*) Patient with Takotsubo cardiomyopathy.** Note high T2 signal and T1 values in an apical slice. (***Right panel***) Patient with regional edema in the anterior wall territory. Note high T2 signal and T1 values in the anterior, anterolateral wall and anterior septum. **Rows:** (**A**) Dark-blood T2-weighted images (reference ROIs in skeletal muscle and remote myocardium not shown). (**B**) Bright-blood T2-weighted images. (**C**) Colour ShMOLLI T1-maps. Green denotes normal myocardium. Red denotes increased T1 values (**D**) R^2^ maps of ShMOLLI inversion recovery fit used to verify the quality of T1-maps. (**E**) LGE imaging.

Compared to controls, patients as a group had significantly higher mean myocardial T1 values, mean myocardial T2 SI _myo:skeletal_ by dark-blood and bright-blood T2w-CMR. There was good correlation between mean myocardial T1 values and T2 SI ratios using dark-blood and bright-blood T2w-CMR (*r* = 0.78 and 0.68, p < 0.001; Figures 1 A and B). There was also good correlation between dark-blood and bright-blood T2w-CMR (*r* = 0.70, p < 0.001; Figure 1C). There was moderate negative correlation between LVEF and mean T1 values (*r* = −0.52, p < 0.001), T2 SI _myo:skeletal_ by dark-blood T2w-CMR (*r* = −0.48, p < 0.002) but not with bright-blood T2w-CMR (*r* = −0.19, p = 0.23).

### Receiver operating characteristics analysis

**Figure 2:**
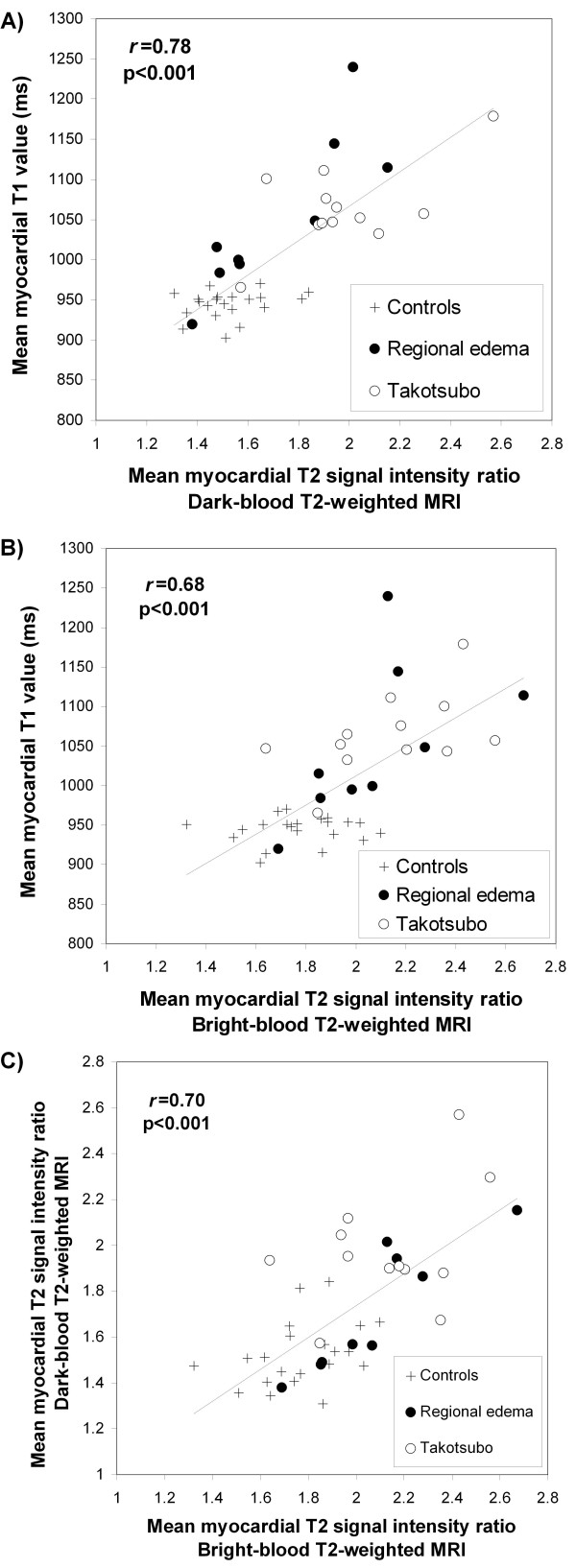
**Relationship between:** (**A**) myocardial T1 values and T2 signal intensity (SI) ratio* derived from dark-blood T2w-CMR; (**B**) **myocardial T1 values and T2 SI ratio derived from bright-blood T2w-CMR;** (**C**) **myocardial T2 SI ratio dark-blood vs. bright-blood T2w-CMR.** *T2 SI ratio = SI_myocardium_/SI_skeletal muscle._

From the 42 subjects, 205 matching slices (111 patient and 94 control slices) across cine, T1-mapping, dark-blood T2 and bright-blood T2 sequences were available (Figure 2). Good coverage of the LV was achieved (median 5 matching short axis slices across all sequences tested, range 3–6 slices per case), yielding 1230 segments.

To maximize the accuracy of the threshold values to detect acute myocardial edema, each segment was strictly assessed for image quality before inclusion into the final ROC analysis. On cine imaging, 21/1230 (1.7 %) segments corresponding to the LVOT were rejected; on T1-maps, 142/1230 (11.5 %) segments were excluded due to off-resonance artifacts, poor T1 fit as evidenced by the R^2^ maps (see Additional file 1), patient movement or low SNR; on dark-blood STIR imaging, 94/1230 (7.6 %) segments were rejected due to insufficient image quality or artifacts; on bright-blood ACUT2E imaging, 26/1230 (2.1 %) segments were rejected due to LVOT anatomy and off-resonance artifacts. This yielded 1005 segments of good quality (453 control and 552 patient segments, 230 of which had abnormal wall motion).

**Figure 3:**
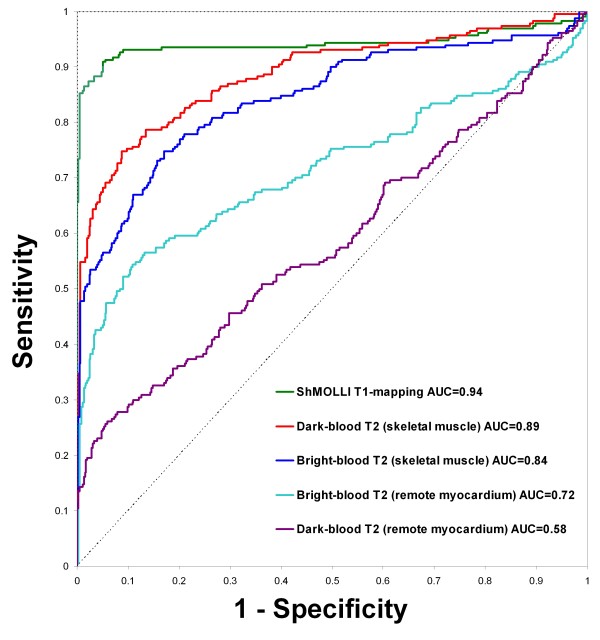
**Receiver operator characteristic curves for the detection of acute myocardial edema by ShMOLLI T1-mapping, dark-blood T2w-CMR and bright-blood T2w-CMR.** For T2-weighted methods, reference regions of interest for comparison of myocardial signal intensity are specified in brackets. AUC = area-under-the-curve.

ROC analysis (Figure 3) showed that for the detection of acute myocardial edema, T1-mapping demonstrated excellent diagnostic performance with a significantly larger area-under-the-curve (AUC = 0.94) compared to dark-blood and bright-blood T2w-CMR, whether the reference ROI was remote myocardium or skeletal muscle (p < 0.04 for all comparisons). A T1 threshold of 990 ms most optimally differentiated segments affected by edema from normal segments, with a sensitivity and specificity of 92 %. This T1 threshold reproduced the proportion of the number of abnormal to control segments used to construct this ROC curve.

We also performed ROC analyses based on patient groups to examine how T1-mapping performed against the T2 methods in detecting regional or global edema when different reference ROIs were used.

**Figure 4:**
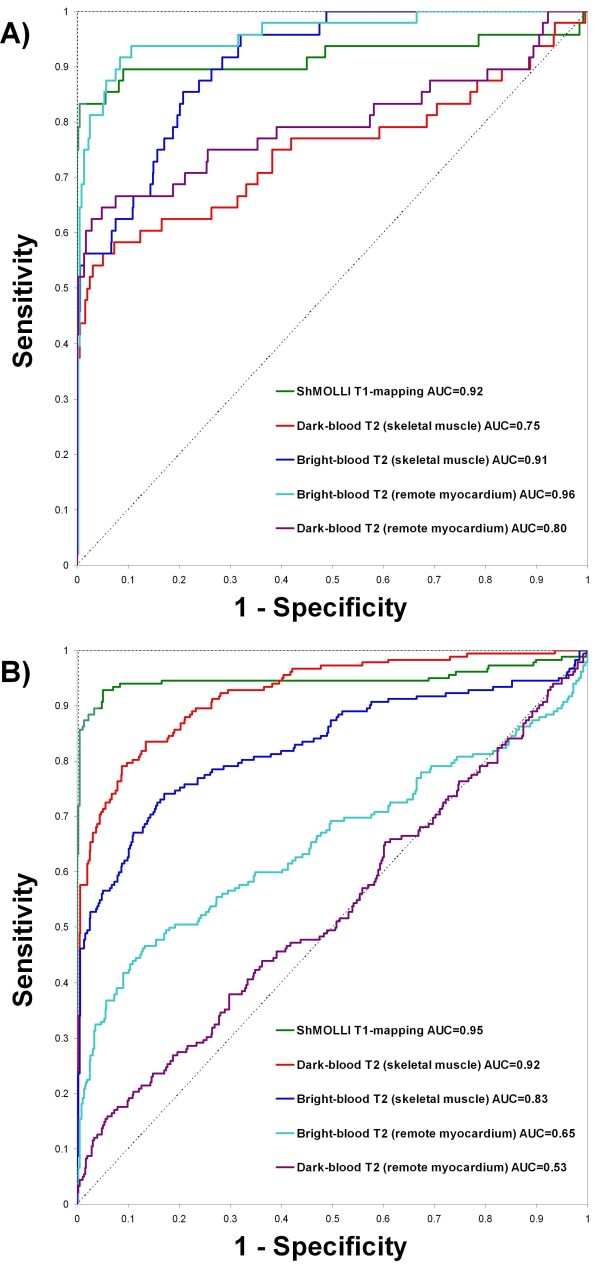
**Receiver operator characteristic curves for the detection of acute myocardial edema according to patient groups using ShMOLLI T1-mapping, dark-blood T2w-CMR and bright-blood T2w-CMR.** (**A**) Patients with regional edema vs. controls. (**B**) Patients with Takotsubo cardiomyopathy vs. controls. AUC = area-under-the-curve.

In acute regional edema, where remote myocardium is more appropriate as a reference ROI for the T2-weighted methods, ROC analysis (Figure 4A) showed that both T1-mapping and bright-blood ACUT2E had equally excellent diagnostic performance (AUC = 0.92 vs. 0.96, respectively, p = 0.21); both were significantly better than dark-blood STIR (AUC = 0.80, p < 0.03 for both comparisons) in the detection of regional edema.

In Takotsubo cardiomyopathy where an entire myocardial slice may be diffusely edematous, skeletal muscle is a more appropriate reference ROI for the T2 methods, and ROC analysis (Figure 4B) showed that both T1-mapping and dark-blood STIR had equally excellent diagnostic performance (AUC = 0.95 vs. 0.92, respectively, p = 0.12) over bright-blood ACUT2E (AUC = 0.83, p < 0.0001 for both comparisons) in the detection of global edema.

**Figure 5:**
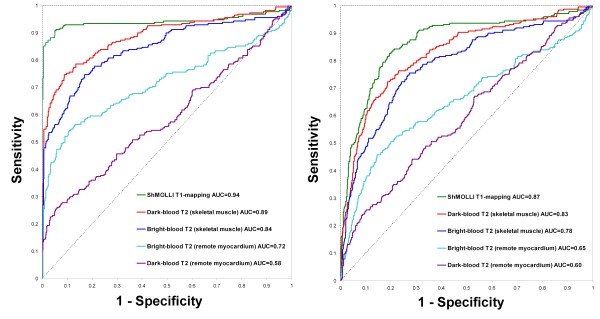
**ROC curves for the detection of acute myocardial edema by ShMOLLI T1-mapping, dark-blood T2w-CMR and bright-blood T2w-CMR using only segments of good quality for all methods (*left panel*), and using all available segments, including segments previously rejected for artifacts for all methods (*right panel.*).** This shows that ShMOLLI T1-mapping retains its superiority despite the re-inclusion of segments affected by artifacts. All comparisons are statistically significant (p < 0.03). AUC = area-under-the-curve.

Finally, to check for bias against any of the methods, when all 1230 segments (including segments affected by artifacts) were used, all of the original relative relationships were preserved, with T1-mapping maintaining a significantly larger AUC of 0.87 compared to the T2-weighted methods (Figure 5). Relative relationships amongst methods were also preserved for ROC analyses according to patient groups (Additional file 1: Figure 6A and B).

### Intra- and inter-observer agreement of T1-mapping

There was excellent correlation (*r* > 0.991) and agreement between T1 estimates obtained between both blinded operators. Bland Altman analysis of intra- and interobserver variability showed that the percentage difference in T1 values was ± 0.5 % (±5.6 ms) and the 95 % CI for the differences was ± 1.1 % of the average T1 (±11.1 ms). There were no statistically significant differences between intra- and interobserver variability.

## Discussion

In this work, we have found that T1 is elevated in regions with abnormal wall motion and that these regions co-localize with regions with elevated T2. There is strong correlation between myocardial T1 and T2 SI ratios. Further, T1-mapping detected myocardial involvement beyond that identified by wall motion abnormality. We have demonstrated for the first time that non-contrast T1-mapping detects acute myocardial edema with high diagnostic accuracy compared to conventional dark-blood and newer bright-blood T2w-CMR at 1.5 T.

### CMR for the detection of acute myocardial edema

Given that edema is a generic tissue response to acute insults, edema imaging appears to be a highly suitable tool for assessing acute myocardial injury. The “gold standard” for defining myocardial edema on imaging, however, is controversial [22]. Experimental animal models using fluorescent microspheres to delineate myocardium area-at-risk in acute MI correlated to T2w-CMR imaging [4]; however, topographic correlation between T2w images and histopathological validation of pure myocardial edema without infarction in humans is lacking, largely due to the difficulty in accurately measuring edema in histopathological samples. We thus decided to study patients presenting with cardiac conditions known to involve acute edema but without co-existing infarction or other pathology detected by LGE. In Takotsubo cardiomyopathy and acute myocardial stunning, inflammation and edema are main features described on human endomyocardial biopsies and animal models [23-25]. Although patients with regional edema did not fulfill criteria for the typical patterns of Takotsubo cardiomyopathy, and possible mechanisms may include coronary spasm or plaque events, the cases precipitated by an emotionally stressful event may represent an atypical form of stress-induced cardiomyopathy not fitting the traditional description of apical ballooning or other morphologic variants commonly described [17]. There was no viral history to suggest a myocarditic process. The common feature for all patients in this study was that all had acute wall motion abnormalities associated with edema without infarction on CMR. While not proven histologically in our cohort, it is highly likely that segments with acute wall motion abnormality would demonstrate features of acute edema. Our assumption was strongly supported by the fact that segments with abnormal wall motion showed numerically higher average T2 SI ratio and T1 values than controls.

### ShMOLLI T1-mapping: a novel method for imaging myocardial edema

We recently developed the ShMOLLI technique for fast T1-mapping [16], which overcomes many of the limitations of both conventional MOLLI T1-mapping and dark-blood T2w-CMR. Compared to MOLLI, ShMOLLI is ~50 % shorter in breath-hold times; it is also heart rate- and T1-independent over a wide range of T1 values, and thus is more accurate in measuring long T1s in edema and at fast heart rates [16]. Compared to dark-blood T2w-CMR , ShMOLLI is robust to tachycardia (shown in simulation and phantoms for rates up to 100 bpm [16] and tested in clinical subjects with sinus tachycardia and atrial fibrillation at ventricular rates of up to 150 bpm; unpublished own data). It does not suffer from signal dropout due to through-plane cardiac motion and breath-holds are considerably shorter.

Bright-blood T2 is excellent for detecting large areas of regional edema when there is obvious normal myocardium to be used as a reference, as shown recently [3] and in this work. The main challenge of extending bright-blood T2 imaging for the detection of diffuse edema was the variability of the image quality of reference skeletal muscle, which was often affected by off-resonance artifacts, bright signals from adjacent fat, bright blood vessels within the muscle or signal dropouts. For this reason, STIR outperformed bright-blood ACUT2E in this setting as STIR images have a more homogenous signal across the field of view, and thus the quality of the skeletal muscle was generally better, more consistent and more reliable. This study suggests that bright-blood T2 may not always be suitable as a replacement method for conventional dark-blood T2w-CMR.

T1-mapping using ShMOLLI has now been shown to be highly sensitive to acute changes in myocardial water characteristics which, up to this point, have been evaluated mainly using T2w-CMR. From a mechanistic point of view, T1-mapping and T2-weighted MRI likely detect acute myocardial injury via distinct, but complementary and overlapping mechanisms. Interestingly, we detected significant T1 elevation even in patient segments with normal wall motion (Table 1), a finding accompanied also by significantly higher T2 SI in segments with normal wall motion in Takotsubo patients (by dark-blood STIR but not bright-blood ACUT2E) as well as in patients with regional edema (by bright-blood ACUT2E but not dark-blood STIR). This suggests that T1-mapping may be the more sensitive technique in detecting acute myocardial changes in both global and regional pathologies, and/or detects myocardial changes beyond that identified by wall motion abnormality and the T2-weighted methods tested. Previous experimental work demonstrated that T1 relaxation times are prolonged in regionally ischemic tissue from dog hearts, and while largely reflective of increased free water content of the ischemic tissue, T1 changes exceeded the changes in water content as the duration of ischemia was increased [13]. The prolongation of T1 values was found to be unrelated to changes in metabolic parameters such as tissue content of oxygen, carbon dioxide, hydrogen ions, lactate, adenosine triphosphate or phosphocreatine [13]. Rather, it was postulated that T1 changes may be related to changes in both total water content as well as the relative amounts of water in intracellular and extracellular compartments. Furthermore, changes in sodium and potassium contents and their distribution may impact on the motional freedom of water protons, contributing to prolongation of T1 values in ischemic tissue [13]. Indeed, both of these processes are part of edema evolution in ischemic injury to the myocardium [1]. Thus, while increased T1 values in acutely injured myocardium may largely reflect water characteristics, there may be additional changes that are more readily detected by T1-mapping and to which T2w methods are less sensitive. The precise nature of what these changes are that prolong T1 values will need to be further investigated, but ShMOLLI T1-mapping is a distinct yet complementary technique to T2w-CMR, which adds to our current repertoire of available sequences for assessing acute myocardial injury.

T1-mapping holds potential to quantify both extent and severity of disease and may provide further insights into conditions where T2w-CMR has proven useful. These include quantifying myocardial salvage in acute MI, determining the age of myocardial injury, diagnosing acute myocarditis and cardiac transplant rejection. It may also be useful in describing subacute conditions where myocardial inflammation is implicated, such as cardiac sarcoidosis [26], amyloidosis, and hypertrophic cardiomyopathy [6]. T1-mapping has also been shown to detect changes in chronic cardiac conditions involving diffuse fibrosis [27-29], which is characterized by increased collagen and aqueous content within the myocardium, leading to interstitial expansion.

Unlike the T2w methods examined, ShMOLLI T1-mapping does not require *a-priori* knowledge of where affected and unaffected myocardium (if any) should be for image analysis; it reports absolute T1 values, obviating the need for a reference ROI, and thus has an excellent performance in different models of edema, whether regional or global. Accordingly, T1-mapping can be useful in pathologies where edema maybe diffuse, patchy or not immediately obvious to the eye. Immediate visualization of the extent of injury may be facilitated by dedicated color schemes based on quantitative thresholds, an example of which is shown in Figure 2.

### Quality and reliability of T1-maps

As our T1-mapping method is novel, we aimed to be as accurate as possible in setting the most optimal cut-off value for detecting edema and thus were stringent on selecting only the best segments (without any or with only minimal artifacts) to include in the analysis. This was one of the reasons for the apparently higher rejection rate for T1-mapping compared to other modalities. T1-maps were assessed for quality and reliability in three ways: visual inspection of the T1-map itself, visual inspection of all seven of the raw T1 images for potential artifacts, and the use of R^2^ maps to ensure that the T1 fit is robust for all segments on the T1-map. The feature of R^2^ maps is described in detail with examples in the Additional file 1, and serve as a more objective way to detect segments with poor T1 fit that may not be obvious to the eye; T2-weighted methods tested in this study, on the other hand, do not have this added feature for quality assurance and so their rejection rate may be underestimated. We consider the fact that ShMOLLI T1-mapping has multiple ways for detecting regions with potentially compromised T1 values a strength of the method which makes it more robust, especially to observer bias. ACUT2E is also a relatively novel method that has been validated in acute MIs [3], but not in detecting global edema or non-ischemic pathologies; sometimes, it is unclear whether darker areas within the myocardium are due to SSFP artifacts (as in Figure 2B, middle panel) or whether it is representative of normal myocardium (as in Figure 2B (right panel) versus signal drop-out/artifact in the inferolateral wall (less likely in this case). These reasons contribute to the apparently higher rejection rate of T1-mapping, but do not reflect that the method is artifact-prone; rather, we were strict on selecting only the best segments for the purpose of establishing T1 thresholds with the greatest accuracy.

As presented in the Results section, to check for bias against any of the methods tested, when we re-included all rejected segments affected by artifacts, it was reassuring to note that all of the original relative relationships on the ROC analysis were preserved, with T1-mapping maintaining its superior diagnostic performance with a significantly larger AUC of 0.87 compared to the T2-weighted methods (Figure 5). Relative relationships amongst methods were also preserved for ROC analyses according to patient groups (Additional file 1: Figure 6A and B).

### Limitations

ShMOLLI T1-mapping sometimes demonstrated typical SSFP susceptibilities to off-resonance artifacts and partial volumes effects, especially in very apical slices. These may be circumvented with frequency shifting and potentially by wide-band SSFP. As artifacts may cause falsely high or low T1 values, it is important to have methods for assessing the quality and robustness of T1-maps, such as those used in this study (see Additional file 1). ShMOLLI underestimates the true T1 value by 4 % but in a consistent manner [16]. Thus, when comparing T1 values measured by different T1-mapping techniques, corrected T1 values and thresholds should be used.

As discussed, it is challenging to establish an in-vivo and histopathological reference gold standard for acute myocardial edema; thus, this study used acute regional wall motion abnormality without evidence of infarction as the best clinical surrogate for edema in a carefully selected study population. Pre-contrast increase in myocardial T1 value is non-specific and may be seen in a number of conditions, including acute myocardial injury, chronic infarct scar [14] and cardiac amyloidosis [30], and thus must be interpreted within the clinical context. Moreover, changes of T1 and T2 within the myocardium are dependent on different mechanisms, but the two are closely related. The precise mechanisms leading to increased T1 relaxation times in acutely injured myocardium remain to be established.

Thirdly, T2-mapping is another technique which may be used to detect edema [31] but was not studied in this work. We did, however, compare T1-mapping to the state-of-the-art bright-blood ACUT2E sequence [10], which has recently been shown to be superior to conventional dark-blood STIR imaging in acute MI [3]. Future work will have to directly compare T1 and T2 mapping techniques.

## Conclusion

ShMOLLI T1-mapping is a novel, quantitative method that we have clinically validated for detecting acute myocardial edema. It has a high diagnostic performance compared to dark-blood and bright-blood T2w-CMR, due to both technical advantages and mechanistic differences. T1-mapping using ShMOLLI holds potential to quantify both extent and severity of disease and may provide further insights into conditions where T2w-CMR has proven useful. We predict wide clinical applicability of this promising technique to study a variety of cardiac conditions in future studies.

## Abbreviations

ACUT2E, Acquisition for Cardiac Unified T2 Edema; bpm, beats per minute; ECG, Electrocardiogram; LGE, Late gadolinium enhancement; LVEF, Left ventricular ejection fraction; LVOT, Left ventricular outflow tract; MI, Myocardial infarction; MOLLI, Modified Look-Locker Inversion Recovery; ShMOLLI, Shortened Modified Look-Locker Inversion Recovery; SNR, Signal-to-noise ratio; SSFP, Steady-state Free Precession; TCM, Takotsubo cardiomyopathy.

## Competing interests

US patent pending 61/387,591: SKP and MDR. *SYSTEMS AND METHODS FOR SHORTENED LOOK LOCKER INVERSION RECOVERY (Sh-MOLLI) CARDIAC GATED MAPPING OF T1.* September 29, 2010.

## Authors’ contributions

VMF: contributed substantially to the conception and design of the study, acquired and analyzed the data and drafted the manuscript; SKP contributed substantially to data analyses and critical revision of the manuscript; EDA contributed to data acquisition and critical revision of the manuscript; JMF contributed to data acquisition and critical revision of the manuscript; TDK, RPC, MGF, MDR have critically revised the manuscript; SN participated in the design and coordination of the study and critically revised the manuscript. All authors read and approved the final manuscript.

## Supplementary Material

Additional file 1 Supplemental Material. **Image Post-processing.** Quality assessment of T1-maps – the use of R2 maps. **Figure 6.** Receiver operator characteristic curves according to patient groups for the detection of acute myocardial edema using all segments, including segments previously rejected for artifacts for all methods. **(A)** Patients with regional edema vs. controls. **(B)** Patients with Takotsubo cardiomyopathy vs. controls. AUC=area under the curve. Click here for file
