# Supplementary material for: Non-contrast T1-mapping detects acute myocardial edema with high diagnostic accuracy: a comparison to T2-weighted cardiovascular magnetic resonance
Source: J Cardiovasc Magn Reson. 2012 Jun 21;14(1):42. doi: 10.1186/1532-429X-14-42 (PMC3424120; doi:10.1186/1532-429X-14-42)
Supplement: Additional file 1 — Supplemental Material. Image Post-processing. Quality assessment of T1-maps – the use of R2 maps. Figure 6. Receiver operator characteristic curves according to patient groups for the detection of acute myocardial edema using all segments, including segments previously rejected for artifacts for all methods. (A) Patients with regional edema vs. controls. (B) Patients with Takotsubo cardiomyopathy vs. controls. AUC=area under the curve. [file 1532-429X-14-42-S1.pdf]

Supplemental Material

Supplemental Methods

Image Post-processing

Quality assessment of T1-maps – the use of R^2 maps

The R^2 map is a pixel-wise indicator of the quality of the T1-map, and is dependent on a number of factors, including SNR, partial volume effects, off-resonance frequency shifts, motion or any other sources of error. These factors are not additive across the image and generally have different impact on R^2. As such, there is no specific R^2 value for a particular error source. Low R^2 may indicate T1 bias or just increased T1 variation.

The R^2 map is a visual aid for detection of artifacts. For instance, a T1-map with T1 values having excellent fit to the T1 recovery curve model will have an R^2 map free of artifacts (see figures below). On the other hand, a T1-map with poor fit to the T1 recovery curve (such as due to breathing or mistriggering during acquisition) will have recognizable regions of dark pixels on the edges of myocardium on its R^2 map (see figures below). Similarly, dark SSFP bands encroaching on the myocardium may also produce artifacts on the T1 map and R^2 map (see figures on next pages). Segments significantly affected by poor T1 fit as evidenced on the R^2 maps were rejected, much like segments affected by artifacts on images acquired using other sequences may be rejected on a visual and qualitative basis.

R^2 maps provide an additional means to identify areas with potentially compromised T1 accuracy. These tools allow critical assessment of the quality of T1-maps generated,

which led to an overall higher rejection rate for T1-mapping compared to other modalities studied. However, even with re-inclusion of all segments affected by artifacts, T1-mapping retained superiority in diagnostic performance. Thus, additional tools to assess for artifacts and inaccuracies serve to increase the reliability of T1-mapping compared to some of the standard CMR modalities.

ShMOLLI T1-mapping - No artifacts

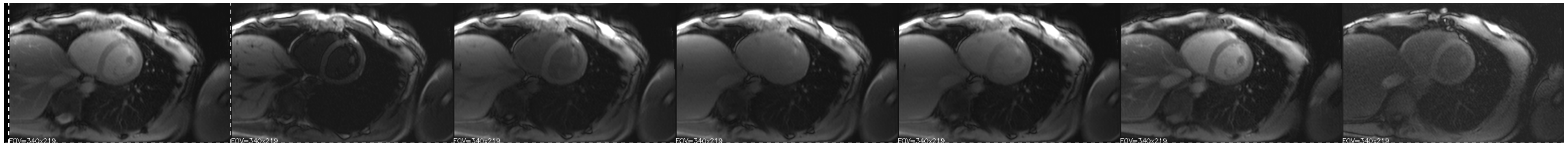

Raw T1 images

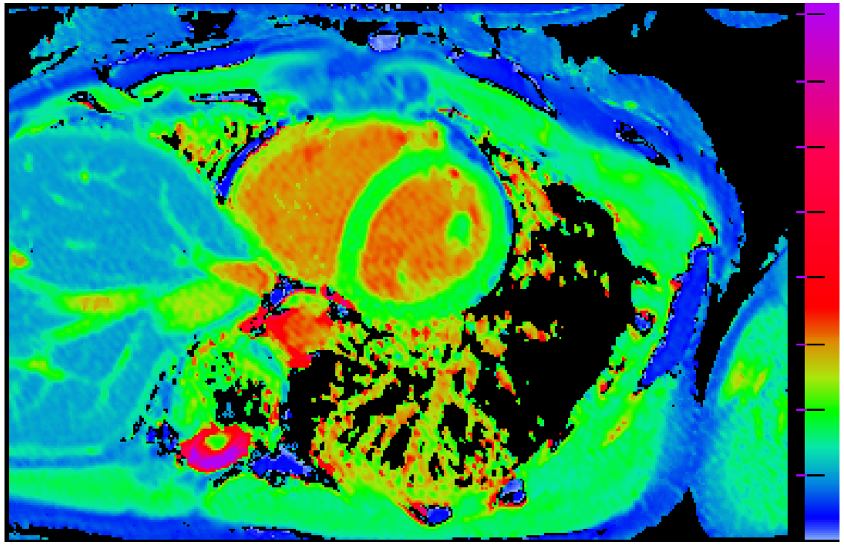

Coloured T1-map

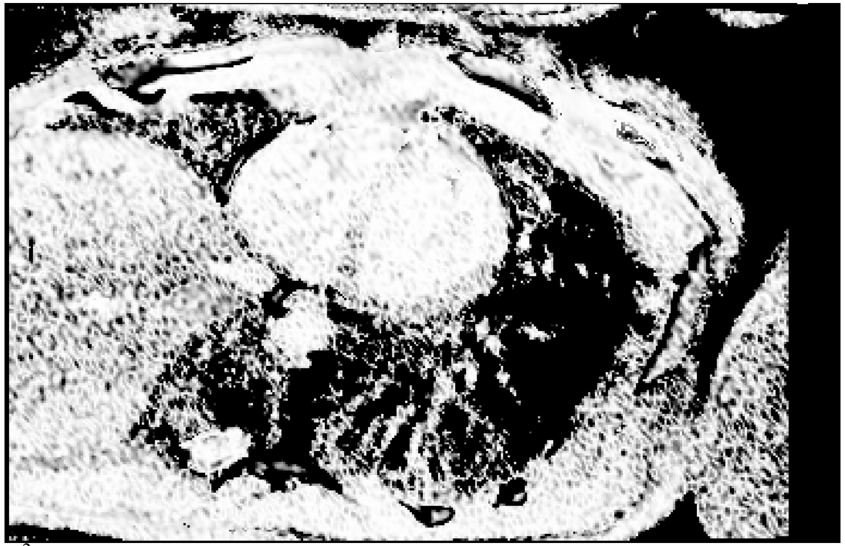

R^2 map

ShMOLLI T1-mapping – Breathing motion artifact

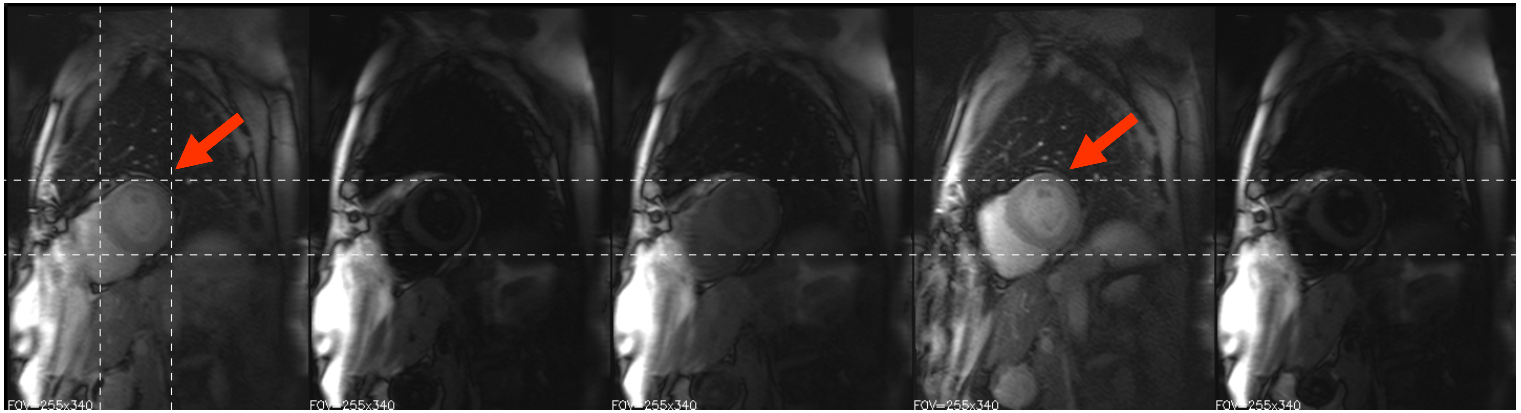

Raw T1 images

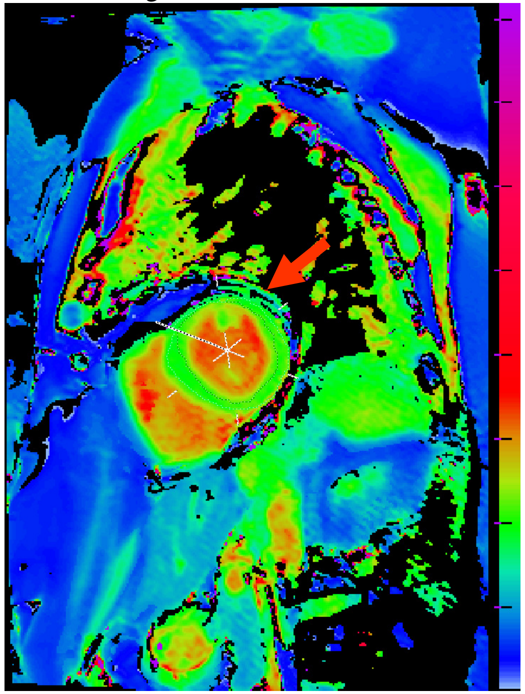

Colored T1-map

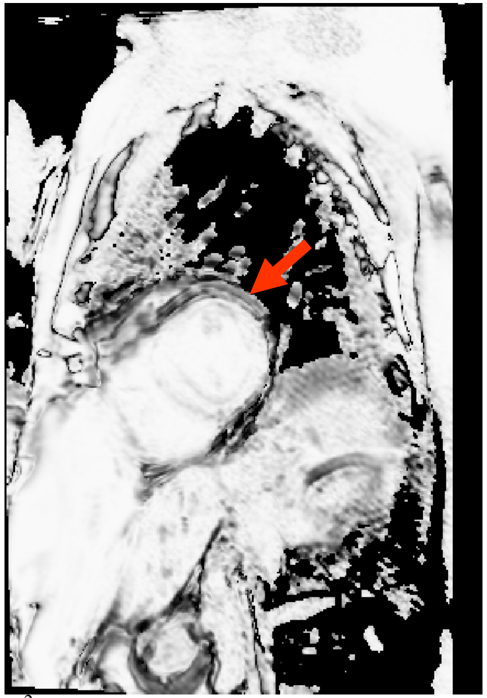

R^2 map

ShMOLLI T1-mapping – Off-frequency artifact (red arrow)

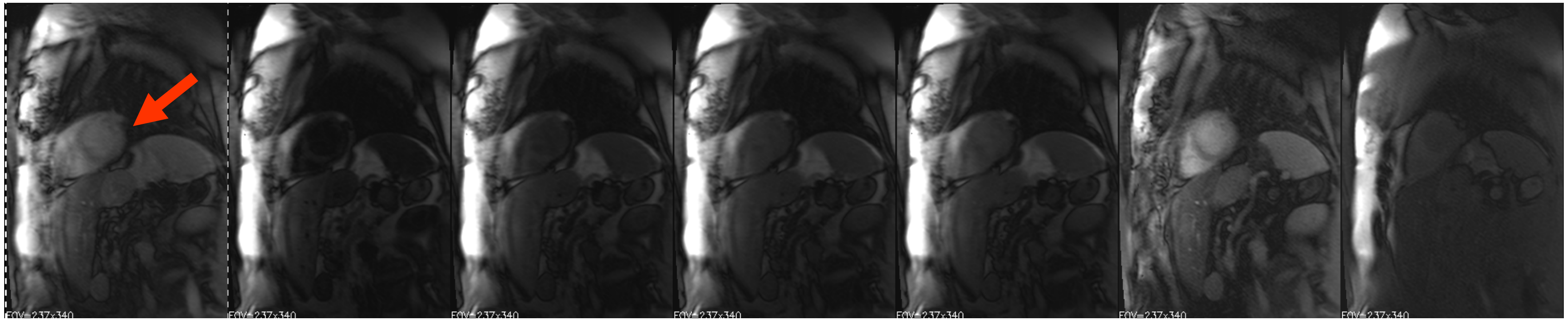

Raw T1 images

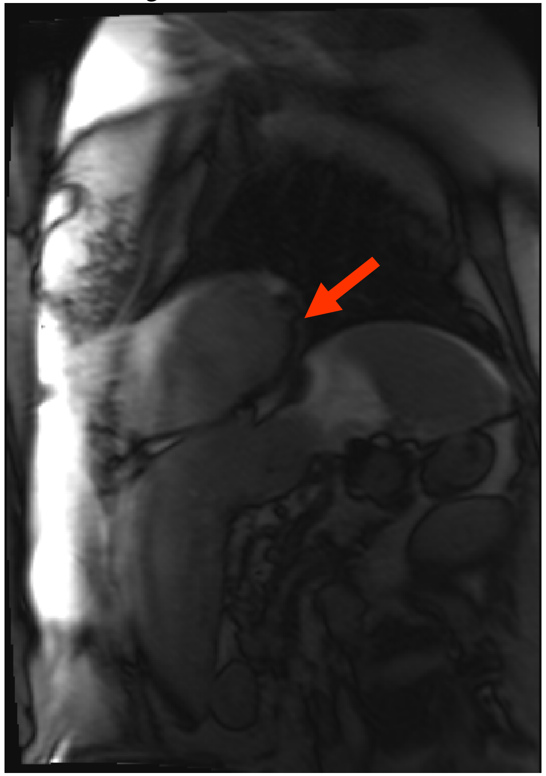

Raw T1 image

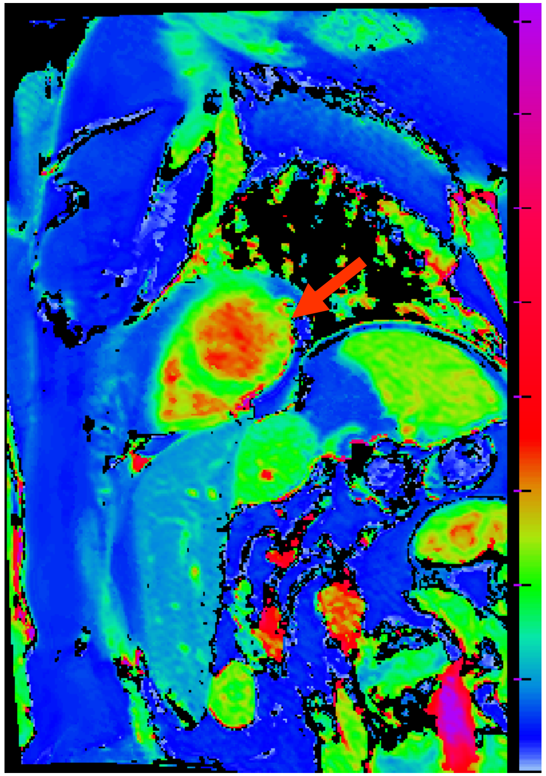

Coloured T1-map

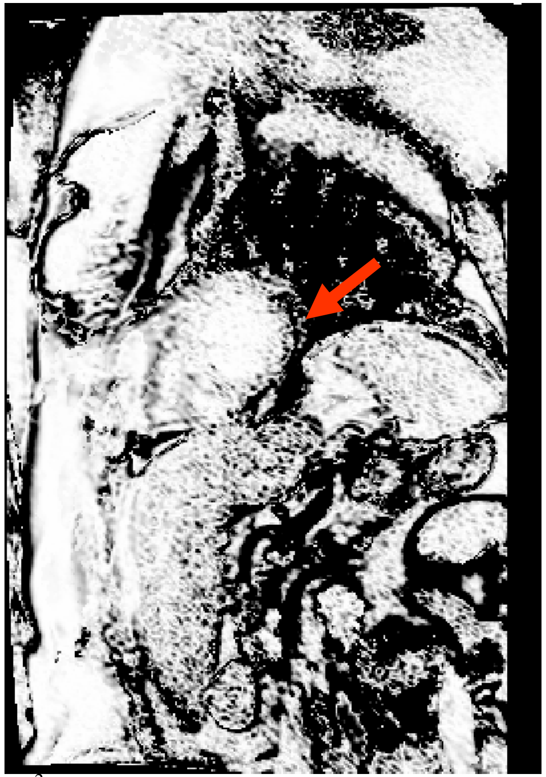

R^2 map

ShMOLLI T1-mapping – Mistriggering during acquisition resulting in poor T1 fit, poor T1-map and poor R2 map

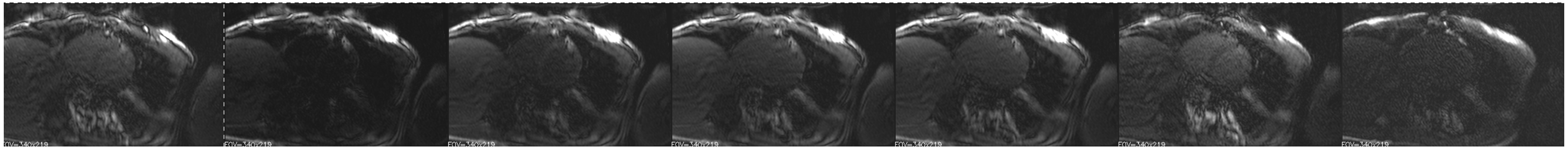

Raw T1 images

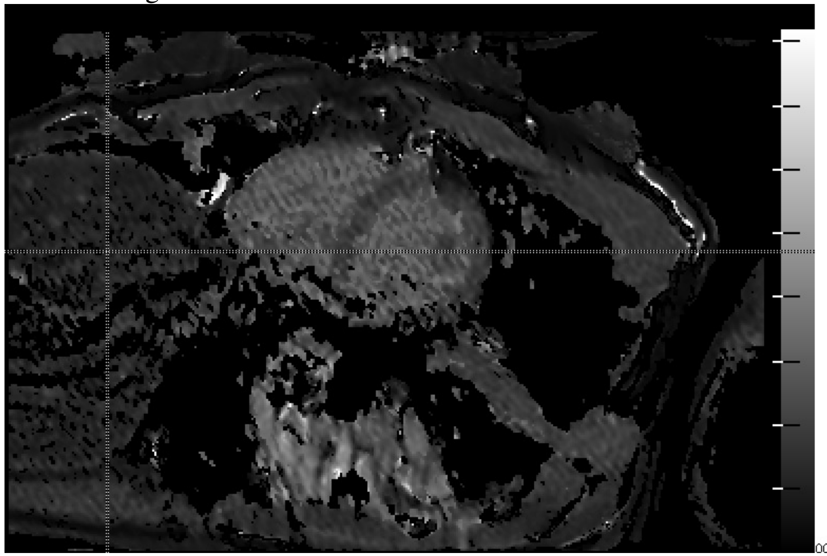

T1-map

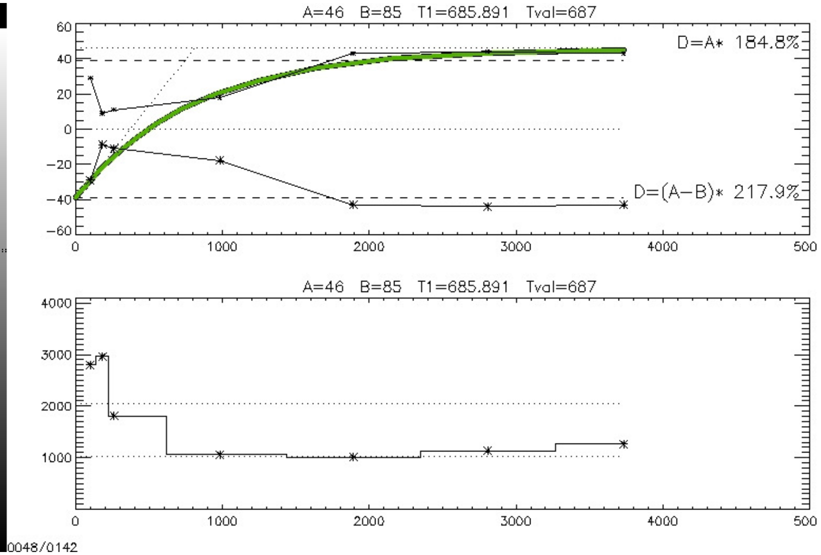

T1 curve fitting

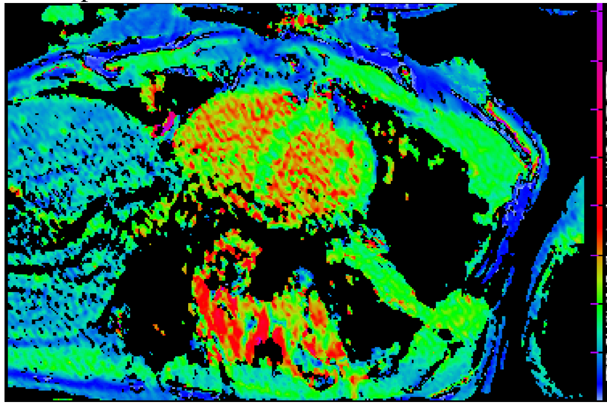

Coloured T1-map

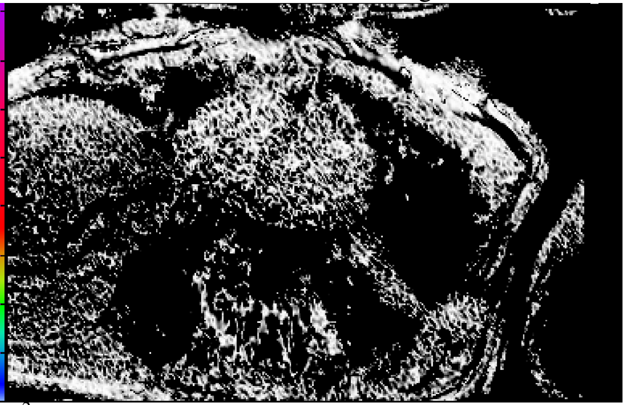

R^2 map

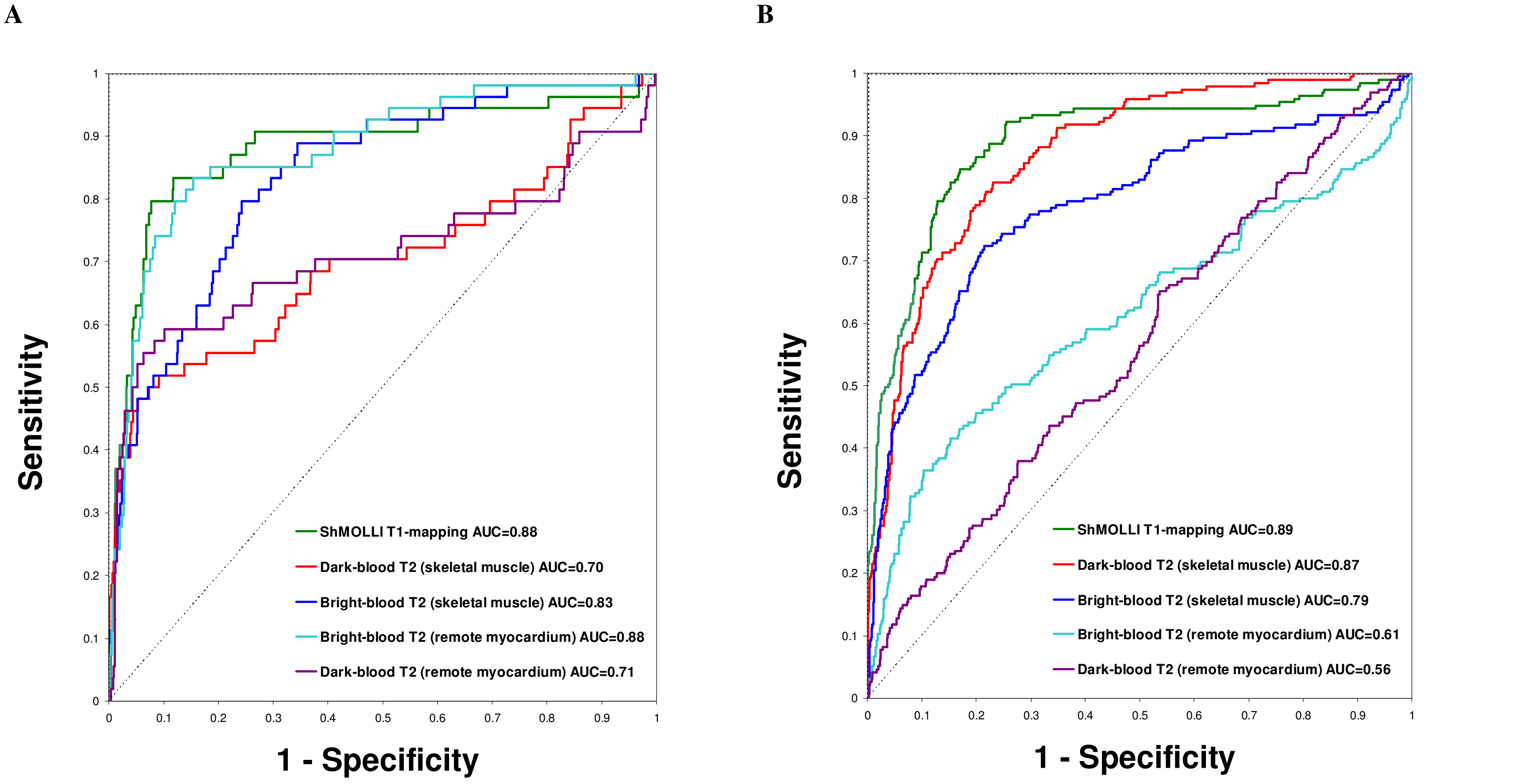

Figure 6. Receiver operator characteristic curves according to patient groups for the detection of acute myocardial edema using all segments, including segments previously rejected for artifacts for all methods. **(A)** Patients with regional edema vs. controls. **(B)** Patients with Takotsubo cardiomyopathy vs. controls. AUC=area under the curve.
